# *De Novo* Histoid Leprosy in an Elderly: A Case Report and Review of the Literature

**DOI:** 10.1155/2012/219421

**Published:** 2012-11-05

**Authors:** Deepak Dimri, Bhawna Sethi, Yogesh Kumar

**Affiliations:** ^1^Department of Dermatology, V.C.S.G. Govternment Medical Sciences and Research Institute, Uttarakhand, 246174 Srinagar, India; ^2^Department of Pathology, V.C.S.G. Govternment Medical Sciences and Research Institute, Pauri Garhwal, Uttarakhand, 246174 Srinagar, India; ^3^Department of Ophthalmology, V.C.S.G. Govternment Medical Sciences and Research Institute, Uttarakhand, 246174 Srinagar, India

## Abstract

Histoid leprosy, an uncommon variant of lepromatous leprosy, develops usually as relapse in lepromatous patients who are on dapsone monotherapy, but uncommonly arises *de novo*. This rare form of the disease, with unique clinical and histopathological features, may pose a diagnostic challenge even to astute clinicians. We report the case that occurred *de novo* in an elderly who presented with small to large lesions all over the body. A fresh focus on histoid leprosy is the primary objective of this paper, especially in the context of the postglobal leprosy elimination era.

## 1. Introduction

Leprosy is a chronic granulomatous disease caused by *Mycobacterium leprae*. It involves skin, peripheral nerves, and nasal mucosa but is capable of affecting any tissue. Histoid leprosy is an uncommon variant of lepromatous leprosy with characteristic clinical, immunologic, and bacteriologic findings. It occurs in lepromatous patients who relapse after dapsone monotherapy in presence of dapsone resistance or at times *de novo*. In addition to lepromatous leprosy (LL), it may occasionally be seen in unstable borderlineand indeterminate leprosygroups. Irregularand inadequatetherapies, as well as resistance to dapsone,and/or mutant organism (histoid bacillus) are responsible factors [1].

## 2. Case Report

**Figure 1:**
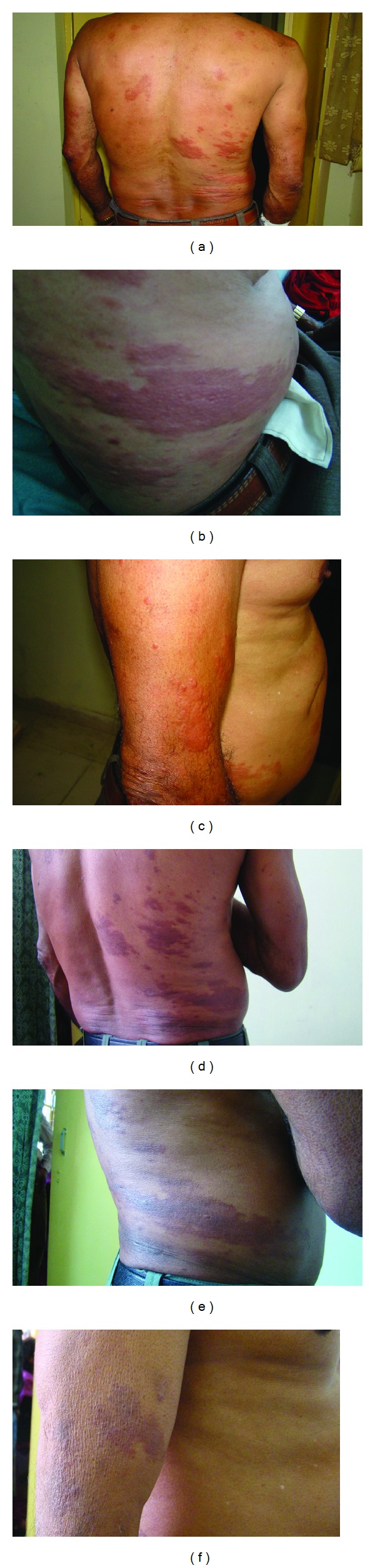
(a-c) Histoid lesions: flesh colored-erythematous papules, nodules, plaques, and patches over back, trunk, arms, and elbows (d&e) Histoid lesions after 6 months' treatment (f) Histoid lesions at 9-month followup.

A 64-year-old farmer presented with shiny, nonitching nodules, papules, and plaques over various parts of body especially prominent over trunk, lower back, buttocks, arms, and legs but involving face, chest wall, elbow, and knee, counting approximately 42 in number (Figures 1(a)–1(c)). The lesions began as papules over anterior abdominal wall and lower back two months back. The lesions were flat-toped, nonscaly, flesh-colored to erythematous, almost symmetrical, normal-oedematous, soft-firm, 0.3 × 0.3 to 10.0 × 5.5 cm in size, painful for past 15 days. Scalp, ears, oral cavity, palms, soles, and genitalia were spared. There was history of low-grade fever for 15 days, associated with redness, pain and discharge from eye, and nasal stuffiness. Nasal bridge was normal. Nerve thickening and lymph node enlargement were absent. There was no impairment of pain, touch, or temperature sensation. No history of epistaxis/insect-bite/trauma/long-term drug intake was given. Family history was noncontributory. Hematological findings were normal except for mild leukocytosis. ELISA for HIV was negative. The clinical considerations included infective diseases (Kala azar/other granulomatous lesions), sarcoidosis, and acute febrile neutrophilic dermatoses.

**Figure 2:**
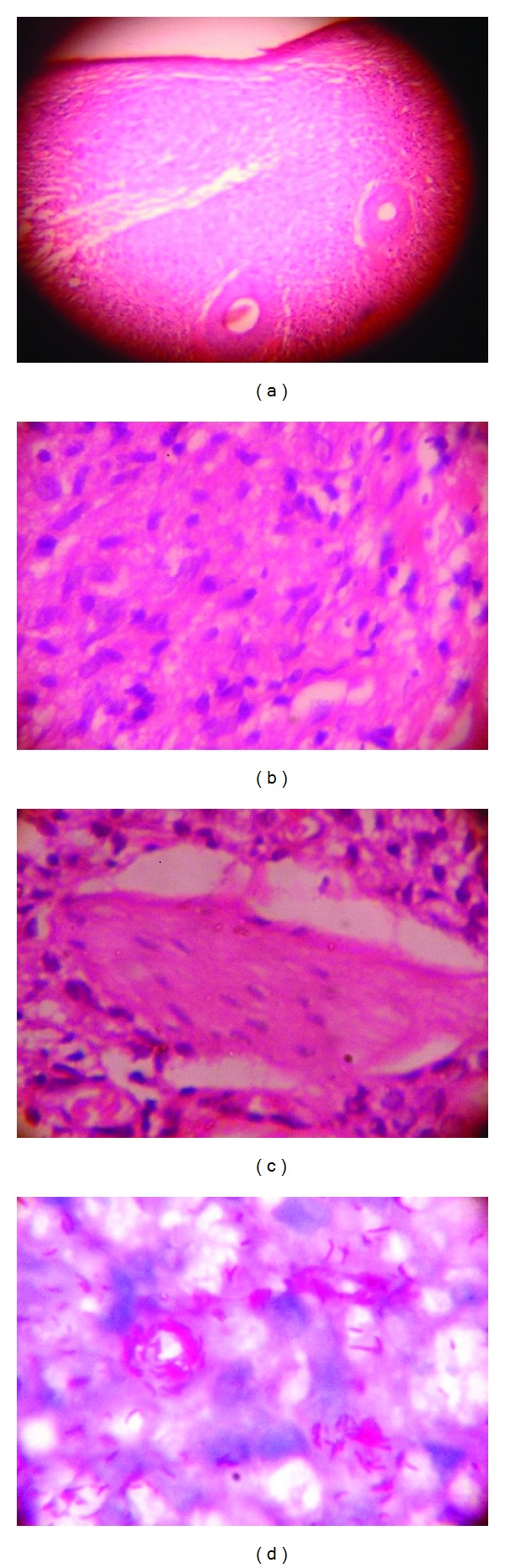
(a) Atrophic epidermis with underlying histoid nodule mimicking benign fibrohistiocytic lesion. Narrow grenz zone and intact hair follicle are appreciable (Leishman, × 40); (b) Leproma at higher magnification depicting spindle-round-polygonal cells (Leishman, × 400); (c) Leproma depicting intact small dermal nerve entrapped within spindle-polygonal vacuolated cells (Leishman, × 400); (d) Myriads of AFB-clusters and singly scattered (Ziehl Neelsen, × 1000).

Histopathology revealed focal epidermal atrophy with an underlying cellular band. The dermis revealed a large, circumscribed collection of benign spindle cells resembling fibroblasts, arranged in a whorled and storiform pattern, with entrapped but preserved small dermal nerve and adenexal structures. Within the mass of spindle-shaped histiocytes, rounded-polygonal plump histiocytic cells with vacuolated cytoplasm along with occasional giant cells were noticed (Figures 2(a)–2(c)). The possibility of benign fibrohistiocytic lesion was kept. However, Ziehl Neelsen (ZN) staining (5% sulphuric acid) revealed abundant, mostly solid, acid fast bacilli (AFB) within the histiocytes and endothelial cells, as well as lying extracellularly (Figure 2(d)). These bacilli were longer than the normal bacilli, scattered singly as well as arranged in clusters. Slit skin smear revealed acid-fast bacilli with bacteriological index (BI) of 4+ to 5+ and microbiological index (MI) of 60–70%. A final diagnosis of *de novo* histoid leprosy was made. The patient has been put on multibacillary multidrug therapy (MBMDT) with rifampicin, clofazamine, and dapsone and is on regular followup. In the past eleven-month treatment, the patient has responded well (Figures 1(d) and 1(e)). 

## 3. Discussion and Review of Literature

Histoid leprosy (HL), initially described by Wade, is considered by some as a variant of lepromatous leprosy (LL),and by others as distinct entity. It is so-called because the microscopic appearance of the nodule shows spindle-shaped cells resembling those in a dermatofibroma. There is male preponderance, and the average age affected is between 21 and 40 years [2]. Although rarely, but it is known to occur at age of ten years or youngerand as old as 84years [1].Our patient was elderly, aged 64. In India, its incidence among leprosy patients has been estimated to be between 2.79 to 3.60% [2].Initial reports were associated with dapsone resistance and with relapse after dapsone monotherapy. However, it has also been reported in patients who relapsed even on supervised monthly dose of multidrug therapy and in patients without any treatment [3]. The histoid leproma can occur during the early stages of LL or borderline LL. In such cases, histoid lesions are transient because the histoid bacilli and the dapsone susceptible *M. leprae* thrive hand in hand initially, but later, the dapsone susceptible bacilli proliferate more than the histoid bacilli. In cases of relapse, the histoid leproma lasts longer because the dapsone susceptible bacilli have been wiped out and the latent histoid bacilli proliferate. The enormous bacillary population in histoid lesions is suggested to be due to focal loss of immunity [4]. HL has characteristic clinical, histopathological, and bacterial morphological features. Clinically it is characterized by multiple discrete shiny, smooth, painless, succulent, globular, protuberant, firm, skin colored to yellow brown, cutaneous, and, or subcutaneous nodules, papules, and plaques, on apparently normal skin. The lesions are usually located on the posterior and lateral aspects of arms, buttocks, thighs, dorsum of hands and on the lower part of the back and over the bony prominences, especially over the elbows and knees [2]. Reports are available with pronounced involvement of the face, the ears may show little change or may be unaffected [1, 5]. In more severely affected cases, mucosal and genital lesions have also been recorded.The palms and soles are usually not affected in histoid leprosy [1]. The peripheral nerves may be thickened [2] or normal [5].Histoid lesions have also been found along the course of the peripheral nerve trunks and cutaneous nerves. The ulnar nerve has been reported as the commonest nerve involved [4].

Lesions vary in size and consistency. The reports reveal usually the size of 1.5–3 cm in maximum dimensions [2, 5, 6], although giant lesions have also been reported [7]. These are usually firm, may be translucent and shiny, with an erythematous or coppery colour. The nodules in our patient felt soft during the excision suggesting probably a recent lesion, whilst the chronic lesions were firmer. Their number may vary from 3–50 [1]. Facies with relics of LL such as infiltration, loss of eyebrows, and depressed nose may be present or absent [1]. Histoid Hansens clinically simulates dermatofibromas, xanthomas, neurofibromas, reticulohistiocytosis, and cutaneous metastasis. It may even masquerade acute sarcoidosis, keloid, molluscum contagiosum, mycobacterial spindle cell pseudotumor, and papulonodular variant of secondary syphilis sparing the palms and soles [1, 6, 8]. Each of them can be differentiated from histoid Hansen on the basis of the characteristic histopathology, absence of mycobacteria on slit skin smear, and nerve thickening.

 Slit skin smear from histoid lesions shows abundant acid fast bacilli occurring in clusters, singly or tightly, packed in macrophages. The bacilli appeared longer with tapering ends, when compared to ordinary lepra bacilli, usually arranged parallel to the long axis of cells. They are considered as mutant bacilli resulting from drug resistance. BI may be 4+ to 6+ and MI may also be high [5, 9], although BI of 0.16 and MI of 0 have also been reported [6].

Histopathological findings are unique. Epidermis may be normal [6], or atrophic due to dermal expansion by the underlying leproma (histoid nodule) and an acellular band (Unna band/grenz zone) located immediately below the epidermis. The leproma consists of fusiform histiocytes arranged in a whorled, criss-cross, or storiform pattern. The spindled histiocytes resemble fibroblasts and it is suggested that these fibroblast-like macrophages may have arisen from tissue histiocytes rather than from blood monocytes [9]. In their most active form, histoid nodules expand rapidly, producing pseudocapsules of compressed collagen at their periphery. These nodules often have central liquefaction necrosis with massive bacillary proliferation and neutrophilic infiltrations, characteristic of local exacerbation reaction [10]. Within the histiocytes, plenty of AFB can be seen. They are longer than the normal bacilli, are uniform in length, more often solid, and are arranged in parallel bundles along the long axis of the spindle histiocytes (histoid habitus) with or without globus formation [1]. There are three histological variants of histoid Hansens, namely, pure fusocellular, fusocellular with epitheloid component, and fusocellular with vacuolated cells. The third pattern is most commonly observed [8]. Histopathological differentials include dermatofibromas and neurofibromas and fibrohistiocytoma.

The aetiopathogenesis of this entity is not clear but an increased cell-mediated (CM) and humoral immunity against *M. leprae* and augmented local CMI demonstrated by necrosis and ulceration have been observed. These findings have been suggested to represent a hyperactive expression of multibacillary leprosy in an effort to restrict/localize or focalize the disease [6].

 Histoid leprosy is managed by initially giving ROM therapy with rifampicin 600 mg, ofloxacin 400 mg, minocycline 200 mg once, which is followed by MDR therapy [2] or MBMDR [9]. An erythema nodosum leprosum (ENL) reaction is known to occur during treatment [11].

Our patient presented with small to large, papules and plaques in the unexposed areas. Ear involvement, nerve thickening, alteration of sensation, previous history, and family history were absent. Associated sign/symptoms were misleading and might be due to flu-like syndrome. The slit skin smear and histopathological examination confirmed the diagnosis of histoid Hansen; a case arising *de novo* in an elderly. The patient is responding to the treatment, with no ENL till 11-month followup.

## 4. Conclusion

As the bacillary load is very high in these patients, they can form a potential reservoir of the infection. It is essential to continue the surveillance for new case, rather than to wait for voluntary reporting since early diagnosis and complete treatment are important to achieve our goal of elimination of leprosy.
